# Understanding reasons and factors for participation and non-participation to a medication adherence program for patients with diabetic kidney disease in Switzerland: a mixed methods study

**DOI:** 10.1186/s13098-022-00898-7

**Published:** 2022-09-27

**Authors:** Carole Bandiera, Liliane Lam, Isabella Locatelli, Jennifer Dotta-Celio, Dina Duarte, Gregoire Wuerzner, Menno Pruijm, Anne Zanchi, Marie P. Schneider

**Affiliations:** 1 School of Pharmaceutical Sciences, University of Geneva, Geneva, Switzerland; 2 Institute of Pharmaceutical Sciences of Western Switzerland, University of Geneva, Geneva, Switzerland; 3 Center for Primary Care and Public Health (Unisanté), University of Lausanne, Lausanne, Switzerland; 4 Service of Nephrology and Hypertension, Department of Medicine, Lausanne University Hospital and University of Lausanne, Lausanne, Switzerland; 5 Service of Endocrinology, Diabetes and Metabolism, Department of Medicine, Lausanne University Hospital and University of Lausanne, Lausanne, Switzerland

**Keywords:** Medication adherence, Electronic adherence monitoring, Patient selection, Patient satisfaction, Patient preference, Diabetes mellitus, Diabetic nephropathies, Chronic renal insufficiency, Interventions, Qualitative research, Interprofessional program, Pharmacists

## Abstract

**Background:**

An interprofessional medication adherence intervention led by pharmacists, combining motivational interviews and feedback with electronic monitor (EM) drug assessment, was offered to all consecutive patients with diabetic kidney disease (DKD) (estimated glomerular filtration rate < 60 mL/min/1.73 m^2^) visiting their nephrologist or endocrinologist. Approximately 73% (202/275) of eligible patients declined to participate, and the factors and reasons for refusal were investigated.

**Methods:**

Sociodemographic and clinical data of included patients and those who refused were collected retrospectively for those who had previously signed the general consent form. Multivariate logistic regression analysis was performed to identify independent variables associated with non-participation. Patients who refused or accepted the adherence study were invited to participate in semi-structured interviews. Verbatim transcription, thematic analysis, and inductive coding were performed.

**Results:**

Patients who refused to participate were older (n = 123, mean age 67.7 years, SD:10.4) than those who accepted (n = 57, mean age 64.0 years, SD:10.0, p = 0.027) and the proportion of women was higher among them than among patients who accepted it (30.9% vs 12.3%, p = 0.007). The time from diabetes diagnosis was longer in patients who refused than in those who accepted (median 14.2 years IQR 6.9–22.7 vs. 8.6 years, IQR 4.5–15.9, p = 0.003). Factors associated with an increased risk of non-participation were female sex (OR 3.8, 95% CI 1.4–10.0, p = 0.007) and the time from diabetes diagnosis (OR 1.05, 95% CI 1.01–1.09, p = 0.019).

The included patients who were interviewed (n = 14) found the interprofessional intervention useful to improve their medication management, support medication literacy, and motivation.

Patients who refused to participate and who were interviewed (n = 16) explained no perceived need, did not agree to use EM, and perceived the study as a burden and shared that the study would have been beneficial if introduced earlier in their therapeutic journey. Other barriers emerged as difficult relationships with healthcare providers, lack of awareness of the pharmacist’s role, and negative perception of clinical research.

**Conclusions:**

Investigating the factors and reasons for participation and non-participation in a study helps tailor intervention designs to the needs of polypharmacy patients. Patients who refused the adherence intervention may not be aware of the benefits of medication management and medication literacy. There is an urgent need to advocate for interprofessional outpatient collaborations to support medication adherence in patients with DKD.

*Trial registration* Clinicaltrials.gov NCT04190251_PANDIA IRIS.

## Background

### Context

In 2021, one in 10 adults worldwide was diagnosed with diabetes of any kind, representing 537 million adults [1]. Preventive measures should be adopted to slow down the pandemic, as it is estimated that 10.2% of the world’s population will be diabetic by 2030. In Switzerland, 500,000 people have diabetes, representing 6% of the Swiss population [2, 3].

Among people diagnosed with diabetes, 30–40% develop chronic kidney disease over time, making it one of the first causes of end-stage renal disease (ESRD) in the occidental world. Treating diabetic kidney disease (DKD) is complex and requires an interprofessional team of healthcare providers (HCP) to improve prognosis, slow down the decline in renal function, and prevent cardiovascular events [4, 5].

Optimal medication adherence is essential to achieve these goals. Medication adherence refers to the extent to which a patient takes a treatment as prescribed. It is characterized by the initiation (first dose of the treatment taken), implementation (dose taken at the right time, with the right regimen, and according to specific requirements), and absence or presence of discontinuation (the patient stopped taking the drug before the end of the prescription). More than 700 determinants characterize medication adherence, including patient-, condition-, treatment-, socioeconomic and health care system-related factors (e.g., patients’ relationship with their HCP) [6, 7].

The risk of ESRD declines if the patient’s adherence to antihypertensive medication is higher than 80% (hazard ratio (HR) 0.67, IC 95% 0.54–0.83) [8]. Although up to 40% of DKD patients encounter medication implementation issues during their care itinerary [9], medication nonadherence is often not addressed by HCPs. Finally, it is estimated that increasing medication adherence in patients with diabetes would lead to annual cost savings of at least US$661 million in the USA [10].

The randomized and controlled PANDIA-IRIS study (*Patients Diabétiques et Insuffisants Rénaux: un programme interdisciplinaire de soutien de l’adhésion thérapeutique*) aimed at monitoring—and if necessary improving— medication adherence among patients diagnosed with DKD who consulted the diabetes and renal outpatient clinics at the Lausanne University Hospital (*Centre Hospitalier Universitaire Vaudois*, CHUV, Lausanne, Switzerland). The intervention included patients in the local Interprofessional Medication Adherence Program (IMAP) at the community pharmacy of the Primary Care and Public Health *Unisanté* (Lausanne, Switzerland) [11]. The program consists of regular motivational face-to-face interviews between the patient and pharmacist to explore patients’ medication management monitored by electronic monitors (EMs) (MEMS and MEMS AS, AARDEX Group, Sion, Switzerland) that capture each date and time the EM is opened by the patient. The protocol has been previously described [12]. This study approached 275 eligible DKD patients, but 73% of the patients declined to participate. This low recruitment success rate seems to be recurrent in the scientific literature; for example, in another study recruiting Type 1 diabetes patients for a randomized controlled trial (RCT), 71% of the patients refused to participate [13]. In another RCT recruiting type 2 diabetes patients, 51% of the patients refused to participate [15]. Such frequent, high levels of patient non-participation and attrition alter the study power and reliability of interventional studies.

Few studies have explored patient factors and reasons for non-participation in interventional programs to promote diabetes self-management education [14–17]. Among the predominant factors cited, the relationship between the patients and their HCP plays a key role in the decision to participate to self-management programs [16]. None of the studies have explored participation in interventions to support medication adherence in patients with DKD.

### Objectives

The primary objective of the PART-PANDIA (*PARTicipation in PANDIA-IRIS*) study was to compare the population of patients with DKD who refused versus accepted to participate in the PANDIA-IRIS study according to their sociodemographic and clinical variables.

The secondary objective was to understand in depth the reasons for non-participation, the patients’ actual medication management process, their relationship with their HCP, and their perception of the role of interprofessional teams, including the pharmacist. The results were put into perspective with qualitative interviews led with patients with DKD included in the PANDIA-IRIS study, to explore their reasons for participation, their perception of the intervention and their reasons for drop-out if any. Recommendations to adapt the study design were provided upon participants’ feedback.

## Methods

### Ethical considerations

The local ethics committee (Vaud, Switzerland) approved the PANDIA-IRIS study in April 2016 (project ID 2016-01674) and the PART-PANDIA study in February 2021 (project-ID 2020-02540).

### The randomized and controlled PANDIA-IRIS study

#### Aim and design of the PANDIA-IRIS study

The PANDIA-IRIS study was a randomized, controlled, and open trial. Enrolled patients were adults diagnosed with diabetes and renal impairment (estimated glomerular filtration rate (eGFR) < 60 mL/min/1.73 m^2^). The included patients were randomized into two arms, each lasting for 24 months—participants in the first arm received IMAP intervention for 6 months vs 12 months in the second arm [12]. During the intervention phase, the patient and pharmacist together explore patients’ medication management habits and skills, patients’ beliefs, preferences and motivation to take the treatment, and patient needs for information regarding the treatment and medication adherence; they investigate the presence of side effects and their management, and they set stepped goals, if necessary, from one interview to the next. The IMAP program is built upon the information-behavior-motivation model of Fisher et al. [11, 18] and contributes to improve patients’ self-management and medication literacy. After the intervention phase (i.e., the follow-up phase), medication adherence was monitored electronically in a blinded manner, without any motivational interviews or feedback. The aim of the PANDIA-IRIS study was to compare medication adherence in both groups to determine the impact of IMAP duration on 24-month medication adherence [12].

#### Patients’ recruitment in the PANDIA-IRIS study

Recruitment began in April 2016, and ended in October 2020. The study recruited 275 eligible patients consecutively (i.e., regardless of the patient’s mother tongue, socioeconomic background, relationship with the HCP, or regularity of attendance to medical appointments). The investigators (JDC and CB) recruited outpatients at the end of their medical consultation at the diabetes and renal outpatient clinics of the Lausanne University Hospital (*Centre Hospitalier Universitaire Vaudois*, CHUV) and at the Center of Primary Care and Public Health *Unisanté*. The physician usually presented the study briefly to the patients before JDC and CB explained the study procedures to the patients in depth. No incentive (e.g., financial) was offered to participants. In total, only 73/275 (27%) patients agreed to participate and were included in the PANDIA-IRIS study.

### The quantitative and qualitative PART-PANDIA study

#### Design of the PART-PANDIA study

**Fig. 1:**
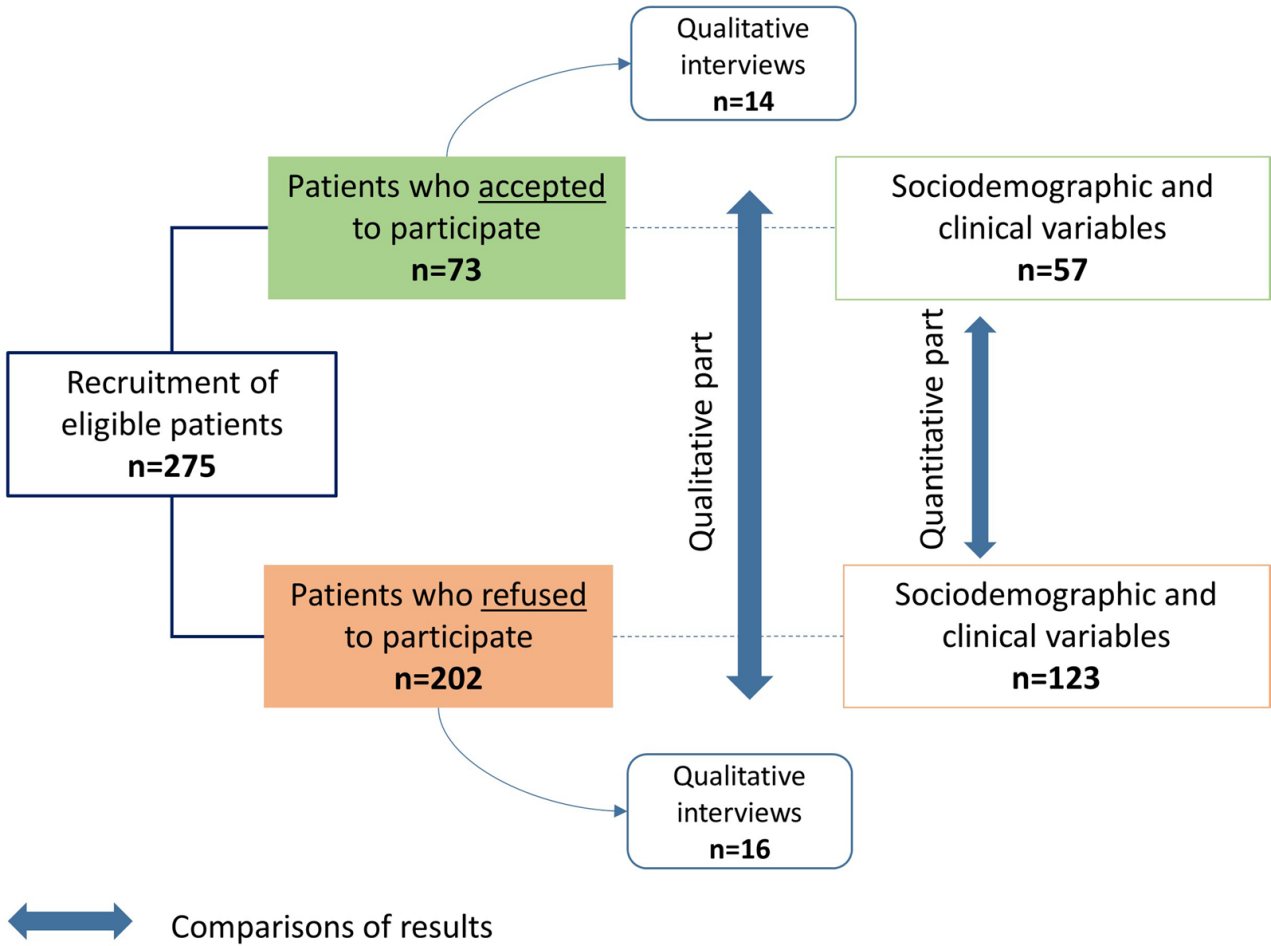
Design of the PART-PANDIA study

The PART-PANDIA study was an observational study with a quantitative and a qualitative part (see Fig. 1). The main ethical and methodological difficulties involved retrieving data from patients who refused to participate. The local ethics committee (Vaud, Switzerland) required that patients who refused participation could be included in the quantitative analysis solely if they had previously agreed to participate and signed the General Consent Form (GCF) of the CHUV. The GCF was offered to all patients who had attended at least one medical appointment at the CHUV. The patient’s agreement with the GCF enables researchers to use their health data as coded (no direct patient identification) for any kind of research project initiated at the CHUV. The ethics committee also agreed to the use of coded health data of deceased patients who did not disagree with the GCF when they were alive. The coded health data of patients who refused to participate in the PANDIA-IRIS study and did not sign the GCF but agreed to sign the specific consent form to participate in the qualitative interview (which specified the use of their health data as coded for this specific study) were also included in the quantitative analysis.

#### Quantitative analysis of sociodemographic and clinical variables

Sociodemographic data (age, sex, civil status, nationality, and patient residence) were collected at the  PANDIA-IRIS study proposal date in each patient’s administrative hospital record. All of the following clinical values measured during the 12 months preceding the PANDIA-IRIS study proposal date were extracted from the patient’s medical record (Soarian®, Cerner) by a data manager through an algorithm for extracting specified data from coded or free-text fields: body mass index (BMI), creatinine blood concentration, eGFR, glycated hemoglobin (HbA1c), low-density lipoprotein (LDL) cholesterol, and systolic and diastolic blood pressure. Each variable was expressed as the 12-month mean value per patient to decrease the risk of outlier values as the frequency of medical appointments—and thus the collection of clinical variables—varies from a patient to another within a year. In case of missing data, discrepancies, or inconsistencies in the database, data were checked individually by the investigator in the patient’s medical record. If no data were available within this timeframe, they were labelled as missing.

The clinical variables collected were used to characterize the population. These variables were also identified as possible risk factors for nonadherence or non-participation to adherence interventions. The following variables were retrieved from patients’ medical records at the medical appointment closest to the recruitment date: diabetes type, number of chronic treatments, time from diabetes diagnosis, cardiovascular events, depression or anxiety diagnosis, smoking status, alcohol or drug addiction, and disability. The missing data were clearly identified. The Strengthening the Reporting of Observational Studies in Epidemiology (STROBE) guidelines were followed to report quantitative data [19].

#### Calculation of the eGFR decline per year

Because renal function declines when diabetes and blood pressure remain uncontrolled [20], we planned to verify if the eGFR decline was different between the included participants and patients who refused inclusion. On the basis of the 2012 Kidney Disease Improving Global Outcomes (KDIGO) guidelines, rapid renal function decline is defined as an eGFR decline of more than 5 mL/min/1.73 m^2^/year [21]. To calculate the patient’s eGFR decline rate per year, all patients’ blood creatinine concentrations available from 2000 to 2021 were extracted from the patients’ medical records. eGFR was calculated according to the Modification of Diet in Renal Disease (MDRD) or, when available, Chronic Kidney Disease Epidemiology Collaboration (CKD-EPI) formula, including sex and race adjustments [22]. If the time span was less than 720 days between the first and last blood creatinine values [23] or if less than four creatinine values were available, the patient was excluded from the analysis.

If a patient received a kidney or any other organ transplant or had undergone a nephrectomy prior to recruitment, patients’ creatinine values were included starting one year post-transplantation or post-nephrectomy and onwards (the kidney function varies significantly during the year following a transplant or nephrectomy). If the transplant or nephrectomy occurred after the recruitment date, or if a patient underwent Renal Replacement Therapy (RRT) or dialysis, patients’ creatinine values were included only until the surgery, RRT, or dialysis date.

Linear regression of eGFR values according to time was performed for each patient [24]. The value of the slope of the linear regression represents the change in eGFR per day, which is multiplied by 365 (number of days in a non-leap year) to obtain the patient’s eGFR decline per year (mL/min/1.73 m^2^/year) (= a). If a ≥ 0, kidney function is stable or increases over time; if a < 0, kidney function decreases.

Linear regression was performed using Microsoft Excel^®^ 32 bites (Microsoft Corporation, Washington) with built-in functions.

#### Statistical analysis

If continuous variables were distributed normally, data were presented as means and standard errors, and Student’s t-test was performed to compare data between groups. If continuous variables were not distributed normally, data were presented as medians and interquartile ranges, and the Wilcoxon-Mann-Whitney test was used to compare data between groups. Categorical variables were presented as numbers and percentages and were compared between groups using the chi-squared test or the Fisher exact test when any expected frequency was less than or equal to 5.

Multivariate logistic regression included only variables showing an association with non-participation in the univariate comparisons (p < 0.2), with a maximum of six variables included (standard rule of thumb—at least 10 events per independent variable in the smallest group size to reduce the risk of overfitting data). The linearity of each continuous variable on the logit scale was tested using the Box-Tidwell test. Statistical analyses were performed using the software for statistics and data science (Stata 17 64 bit) [25].

#### Qualitative interviews with included participants and with patients who refused to participate to the PANDIA-IRIS study

One-to-one interviews with a representative subset of participants were conducted from July 2017 to August 2020. One-to-one post hoc interviews were conducted with a subset of persons with DKD who refused to participate in the PANDIA-IRIS study but accepted the interview afterwards from March to May 2021.

All participants who attended a 30-min interview signed a specific consent form and were compensated with 20 Swiss francs to cover associated costs, such as transport and parking. The interviews were conducted face-to-face at the Lausanne University Hospital, either by JDC or CB. They led the interviews with a semi-structured guide which enabled the investigators to ask planned questions and the patients to explain themselves in depth as well as to address relevant yet unplanned subjects.

The guide for the interviews with PANDIA-IRIS participants was structured with questions exploring patients’ reasons for participation, IMAP intervention sessions with the pharmacist, patients’ perceived positive and negative aspects of the PANDIA-IRIS intervention, and their relationship with HCPs (pharmacists, physicians, and nurses) during the intervention phase.

The interview guide with persons who refused to participate was structured with questions about the history of medication (self-)management (present and past), reasons for study refusal, patient’s relationship with the HCPs, patient’s perceived usefulness and uselessness of the PANDIA-IRIS study, and clinical research in general.

The interviews were audio-recorded and transcribed verbatim using Microsoft Office Word® software. Verbatims were coded by CB and LL using the MaxQDA software® (version 2018). A thematic analysis was performed, and coding was performed using an inductive approach (i.e., codes are driven from raw data to enable the possible emergence of new codes that do not necessarily fit the researcher code preconception) [26]. Themes and categories emerge from the coding process. Each code was compared, and discrepancies were discussed between the investigators to reach a consensus. Data saturation (i.e., no emergence of new codes) was achieved.

To interview a heterogeneous population of patients who agreed to participate, patients from both PANDIA-IRIS arms were interviewed, some of whom had just finished the intervention phase but were pursuing the follow-up phase, some completed the 24-month follow-up, whereas others dropped out prematurely for personal reasons. We included patients with different levels of medication adherence (based on the results of electronic monitoring), sex, and age.

Recruitment of patients who refused to participate was intentionally heterogeneous regarding age, sex, marital status, and renal impairment level (eGFR > or < 50 ml/min/1.73 m^2^). This study followed the Standards for Reporting Qualitative Research (SRQR) guidelines [27].

## Results

### Quantitative part: comparison of sociodemographic and clinical variables of patients who refused versus agreed to participate in PANDIA-IRIS

Among the 275 patients to whom the PANDIA-IRIS study was presented, 169 (61.5%) patients had signed the GCF, six patients died and did not disagree with the GCF while they were alive, and five patients signed the specific consent form allowing data retrieval and participated in the qualitative interview. In total, the sociodemographic and clinical data of 180 patients (123 patients who refused and 57 who accepted) were included in the quantitative analysis. According to their GCF endorsements, patients included in the PANDIA-IRIS study were more willing to participate in research projects (57/73, 78%) than those who refused participation in PANDIA-IRIS (112/202, 55%), p = 0.0007.

The sociodemographic and clinical variables of the patients who refused versus agreed to participate in the PANDIA-IRIS study are presented in Table 1. Patients who refused to participate were older (mean age 67.7 years, SD:10.4) than those who accepted (64.0 (SD:10.0), p = 0.027). The percentage of women was higher among patients who refused than among those who accepted it (30.9% vs 12.3%, p = 0.007). The time from diabetes diagnosis was longer in patients who refused than in those who accepted (median 14.2 years, IQR 6.9–22.7 vs 8.6 years, IQR 4.5–15.9, p = 0.003) (Table 1). In the multivariable logistic regression analysis, factors associated with an increased risk of non-participation were female sex (OR 3.8, 95% CI 1.4–10.0, p = 0.007) and the time from diabetes diagnosis (OR 1.05, 95% CI 1.01–1.09, p = 0.019) (Table 2).

**Table 1:** Sociodemographic and clinical variables of patients with diabetic kidney disease eligible to the PANDIA-IRIS study

	Patients who accepted n = 57	Patients who refused n = 123	p-value
Sociodemographic variables			
Age (years), mean (SD)	64.0 (10.0)	67.7 (10.4)	P = 0.027
Female, n (%)	7 (12.3)	38 (30.9)	P = 0.007
Married/partnership civil status^a^, n (%)	26 (45.6)	66 (53.7)	P = 0.315
Swiss nationality, n (%)	35 (61.4)	91 (74.0)	P = 0.087
Patients living in the Lausanne center or surroundings (maximal distance of 20 km), n (%)	39 (68.4)	88 (71.5)	P = 0.669
Clinical variables			
Type 2 diabetes^b^, n (%)	54 (94.7)	108 (87.8)	P = 0.149
Body Mass Index (BMI), median (IQR)	31 [28–34]	29 [26–32]	P = 0.101
Creatinine blood concentration (*µ*mol/l), median (IQR)	128.7 (100.7–154.5)	123.4 (95.5–157.5)	P = 0.465
eGFR (mL/min/1.73m^2^), median (IQR)	49.0 (37.0–60.0)	48.8 (34.3–61.0)	P = 0.876
eGFR decline per year (mL/min/1.73m^2^/year), median (IQR)	−2.4 (−4.2; −0.7)	−1.8 (−4.2; −0.5)	P = 0.431
HbA1c (%), median (IQR)	7.1 (6.7–8.0)	7.4 (6.8–8.3)	P = 0.228
LDL-cholesterol (mmol/l), mean (SD)	2.1 (0.7)	1.9 (1.0)	P = 0.263
Systolic blood pressure (mmHg), mean (SD)	135.8 (15.5)	137.8 (15.3)	P = 0.415
Diastolic blood pressure (mmHg), mean (SD)	76.9 (8.8)	74.2 (9.5)	P = 0.065
Number of oral prescribed chronic treatments at the time of recruitment, mean (SD)	9 [4]	10 [3]	P = 0.228
Time from diabetes diagnosis (years), median (IQR)	8.6 (4.5–15.9)	14.2 (6.9–22.7)	P = 0.003
Current or past cardiovascular event(s)^c^, n (%)	12 (21.1)	32 (26.0)	P = 0.471
Depression or anxiety diagnosis, n (%)	11 (19.3)	18 (14.6)	P = 0.428
Current smoker at the time of recruitment, n (%)	9 (15.8)	16 (13.0)	P = 0.616
Current or past alcohol addiction, n (%)	17 (29.8)	33 (26.8)	P = 0.676
Current or past drug addiction, n (%)	1 (1.8)	3 (2.4)	NA
Disability or amputation or handicap, n (%)	3 (5.3)	15 (12.2)	P = 0.149

NB1: Pearson’s chi-squared test was used for the following variables; Female sex, Married/partnership civil status, Swiss nationality, Patients living in the Lausanne center or surroundings, Type 2 diabetes, Current or past cardiovascular event(s), Depression or anxiety diagnosis, Current smoker at the time of recruitment, Current or past alcohol addiction, Disability or amputation or handicap; Fischer’s exact test was used for current or past drug addiction; T-Student test was used for: age, LDL-cholesterol, Systolic and diastolic blood pressure, Number of oral prescribed chronic treatments; Mann–Whitney test was used for: BMI, Creatinine blood concentration, eGFR, eGFR decline per year, HbA1c, Time from diabetes diagnosis

NB2: among patients who refused participation, missing data were: 2 BMI values, 6 creatinine blood concentrations, 6 eGFR values, 5 eGFR decline per year values, 34 HbA1c values, 39 LDL-cholesterol values and 10 values for the time from diabetes diagnosis. In patients who accepted, missing data were 11 HbA1c values and 14 LDL-cholesterol values

NA= “not applicable”; statistical test not applicable as the number of patients is too small

^a^The other patients are separated, divorced, widowed or single

^b^The other category includes patient diagnosed with diabetes type 1, glucocorticoid-inducted, post-transplantation or post-pancreatectomy diabetes or Latent Autoimmune Diabetes in Adults (LADA). The eligibility criteria were expanded from October 2019 to include other types of diabetes than type 2, which explains the low proportion of patients in these categories

^c^Stroke, ST elevation myocardial infarction (STEMI) or Non-ST elevation myocardial infarction (NSTEMI), transient ischemic attacks (TIAs), cardiopulmonary arrest

**Table 2:** Multivariate logistic regressions including maximum 6 variables associated with non-participation in the univariate comparisons (p < 0.2)

	OR	95% CI		p-value
Age (years)	1.03	0.99	1.06	0.161
Sex female	3.80	1.44	10.01	0.007
Swiss nationality	1.46	0.69	3.06	0.320
Diastolic blood pressure (mmHg)	0.99	0.95	1.03	0.490
Time from diabetes diagnosis (years)	1.05	1.01	1.09	0.019
BMI	0.96	0.90	1.03	0.236

Patients who agreed to participate took a mean of 9 (SD:4) chronic treatments, whereas patients who refused took 10 (SD:3) treatments (Table 1).

For the included patients, the eGFR decline was calculated based on the mean time span between blood creatinine values of 3030 days (min 729 days, max 4353 days) and the mean number of creatinine values per patient of 65 (min 5; max 192). In patients who refused to participate, the mean time span was 3204 days (min 960 days; max 4342 days), and the mean number of creatinine values per patient was 62 (min 7; max 263). The eGFR decline per year was not significantly different between patients who refused and those who accepted (respective median −1.8 mL/min/1.73m^2^/year (IQR: −4.2; −0.5) vs −2.4mL/min/1.73m^2^/year (IQR −4.2; −0.7) (Table 1). The results show that patients are faster decliners than the average found in type 2 diabetes patients in Swiss ambulatory care (i.e., −1.2 mL/min/1.73m^2^/year (SD 0.05) in men, −1.0 mL/min/1.73m^2^/year (SD 0.06) in women [28]).

### Qualitative part: Interviews on satisfaction with patients included in the PANDIA-IRIS study

Data saturation was achieved after 12/14 interviews. Based on the transcribed verbatim from the 14 interviews, 1182 segments were coded and gathered into the following main themes: (i) reasons for participation, (ii) usefulness of the PANDIA-IRIS intervention and (iii) adaptations of the intervention.

#### Why did patients accept to participate to the PANDIA-IRIS study?

Patients were willing to participate to “improve” medical and clinical knowledge, and therefore “help” other patients (P01, P06, P10, P11, P13). They also wanted to benefit from the intervention to improve their medication management and knowledge about their medicines (P02, P14, P07). Some patients accepted to participate following the recommendation of their endocrinologist or nephrologist (P03, P04, P08, P09, P12).

“I participated because the nephrologist told me that it would be useful for me. It should reassure the doctors that I'm taking my medication regularly.” (P09).

Moreover, patients participated as they were reassured by being monitored clinically throughout the study, in addition to the usual medical appointments (i.e., blood pressure was controlled at each pharmacy visit) (P10, P05, P13). Meeting the pharmacist regularly allows the patient to refer to suspicious symptoms without waiting for the next medical appointment.

“I had a better follow-up [by being included in the study]. It allows us to free ourselves from our health worries.” (P13).

#### How do the participants perceive the usefulness of the PANDIA-IRIS intervention?

- The intervention allows participants to reflect on their own medication management
  The interviewees explained that the intervention was useful and well designed (EM refill, interviews with pharmacists, EM feedback, and adherence reports sent to the physician).
  “*I am very glad I participated, it helped me in my daily medication management (…) I better understand if I take the medication or not or if I take it twice or if I take the right one; so it allows me to be aware. (…) It allowed me to try to adapt, to take it [the medication] better in fact. (…) So I paid more attention to what I had to do.*” (P11).
  Feedback on medication adherence was provided to the patients at each encounter through EM chronology graph-based interviews. Patients explained that the repeated feedback components, based on communication and monitoring, increase their self-reflection and awareness of the way they self-manage their medication (e.g., shed light on forgetfulness and confusion). It decreases the medication burden (e.g., “*If we have an interview, we can talk about it [medication intake] directly, openly, instead of keeping everything in your head*” (P13)).
  The electronic monitors are memory aids to check daily drug intake through a liquid–crystal display (LCD) screen on top of the bottle cap. On study completion, some patients decided to pursue the IMAP intervention in order to continue using EM and to benefit from an interview with the pharmacist.
  *“Before using the electronic monitors, I used to take my medication at 11:00 a.m. and sometimes at 8:00 a.m. When I use the electronic monitor, I am more careful. It makes me aware that I have to be careful. I take them [the medications] more regularly now [since the inclusion in the study]. (…) I have control over my medication intake. Besides, I better understand the role of each medication.”* (P13).
  *“Having a double check of the correct medication taking was useful. It's a relief. (…) I can see what was really done. And I like to see the graph. (…) I realized that I would really like to keep using the electronic monitor [at study completion]”* (P11).
  In addition, after the intervention phase (i.e., during the follow-up phase when the patient used EM without any feedback or intervention), some patients were disappointed not to attend the motivational interviews.
  *“[In the follow-up period], I missed that [the graphic]! Having a discussion and getting feedback and being able to explain our behavior was missing. I think everyone needs feedback*.” (P13).
  In contrast, some patients did not perceive a significant impact of the intervention on their medication routine as they reported managing their medication well.
  “*I don't think [the study] had a huge influence [on the medication management]. It was interesting to see the graph together [with the pharmacist], to analyze if I had forgotten a dose and why and what happened. But I don't think it had a strong impact on how I took them [the medications].”* (P14).
  *“I still took the same medicines, at the same frequency, in the morning, in the evening. It [the intervention] did not make any difference to me. They [pharmacists] never asked me to change my behavior. (…) An electronic pillbox is practical.”* (P05).
- Included patients acquired knowledge about their medication

Pharmacists’ active listening allowed patients to ask their questions about their treatments or disease and to gain additional knowledge (e.g., taking the drug in a fasting condition or not, what to do in the case of a missed dose). The patients appreciated the quality and relevance of the information provided, in addition to the information provided by the physician. The support and clinical skills of pharmacists were appreciated.

*“Sometimes, we don't precisely know what medication we are taking… We talk about this with the pharmacist during the interviews, we can ask questions about medications, what their effects or side effects are.”* (P14).

The newly acquired knowledge complements personal beliefs, which keep conveying ambivalence in certain patients, for example, about the usefulness of treatments in case of frequent changes (“*My medication changes too much. (…) In the long term, a medication is no longer efficient*” (P12)). Some discussed their own use of alternative medicines (“*… nux-vomica, which drains the substrates of the drugs*.” (P08)).

- The adherence report promotes interprofessionnality

Sharing the adherence report with different HCPs was appreciated by the patients. One patient pointed out that it helps to harmonize decisions (e.g., “*My physician agrees with what my pharmacist does*” (P04)). The adherence report is the starting point for physicians to adjust the treatment based on the patient's adherence. One patient (P04) reported that his relationship with his physician improved by participating in the study, as his physician did not have confidence in his medication management before study inclusion.

*“To my mind, the more information the doctor has, the better he can figure it out. Every person is different, every person reacts differently, especially with diabetes. You can't make a general rule.” (P14).*

#### According to participants, what are the intervention adaptations and improvements to be made to the PANDIA-IRIS study design?

The study burden should be as low as possible. Flexibility in the frequency of appointments should be considered (e.g., especially for full-time employed patients) (P03, P04, P10, P14). Patients were already busy managing their medical appointments and felt overwhelmed by adding other pharmacy visits to their routine clinical care (P04, P06, and P10).

Developing interventions in the patients’ usual pharmacies would have been appreciated by some patients (P06, P08). Considering flexibility and adaptations regarding the use of the adherence monitor (i.e., another tool than the EM, as an electronic weekly pill-organizer) may lead to improve recruitment. Indeed, the plurality of treatments requires the use of several EM (P05), and frequent changes in treatments require additional pharmacy visits to adapt EMs’ content (P12), which was reported as a reason for drop-out. In some cases, the use of EM in parallel with a weekly pillbox (P01, P13) disrupts the usual medication management routine and was also a reason for drop-out.

Nevertheless, the 14 patients interviewed unanimously recommended the PANDIA-IRIS study to other patients.

### Qualitative part: interviews with patients who refused to participate

Among the 202 patients who refused to participate, 26 were invited to participate in the qualitative interview and 16 agreed (62%). The main reasons for refusal were a lack of interest in the research and living too far from the hospital to attend the interview.

Data saturation was achieved after 13/16 interviews. Based on the transcribed verbatim from the 16 interviews, 1830 segments were coded and grouped into the following main themes: (i) main reasons for non-participation in the PANDIA-IRIS study, (ii) current and past treatment management at home, (iii) relationship with HCPs, and (iv) perceived HCPs' roles.

#### Main reasons for non-participation in the PANDIA-IRIS study

The reasons for refusal were complex and multifactorial; they involved both patients and their relatives.

- Patients who refused did not feel the need to participate in a medication adherence study, and they did not perceive a sense of medication adherence support.

Patients were confident about their medication management and felt that they would not benefit from such support and were not interested in learning more about their treatment. Some did not even understand the purpose and usefulness of such a support program to the point that they did not consider it at all. Most patients indicated that the intervention program could be useful for more vulnerable or older people (patients with poor knowledge, suffering from cognitive disorders, or depression).

“*As long as I'm not totally senile, I think I'm responsible enough to take all the medications prescribed to me without forgetting any.*” (P15).

*“For me that’s useless to talk about that [treatment management]. (…) I think [the doctor] has other things to worry about than hearing my whining about my medication management (…). I prefer to discuss my illness with the doctor, a more serious thing than my medication management.” *(P08).

- The electronic monitor (EM) was rejected by many patients

Patients did not want to change their medication management habits as they were used to other adherence tools that they chose (e.g., weekly pillboxes, boxes, bags, kits, and small cups), which were perceived as reliable, useful, and convenient. Some patients explained that the EM was too bulky, not easy to carry along, and not suited to manual dexterity impairments. Moreover, some patients explained that they were not confident about the use of electronic tools.

“*It's a routine, every Saturday morning, after breakfast, we [the patient and his wife] prepare the weekly pillbox for the coming week, and there is no problem.”* (P03).

*“Not practical for travelling because […] I will need 7 [electronic monitors], which are still much more cumbersome than if I take my small weekly pillbox to move around.”* (P02).

- The design of the intervention was a burden for patients

Intervention was perceived as an additional burden on patients’ busy medical care. Although scheduled on the same day as the visit to the physician, most of the patients felt overwhelmed by the frequency of appointments at the pharmacy (monthly during the first 3 months, then once every three months). The fact that the intervention could be delivered only in a single academic pharmacy was a barrier to participation, as patients were bound to their usual pharmacy located at a short distance from home, where they were familiar with the staff.

*“There is a logistical issue: I don’t live in the city so I don’t want to come to the city once per month.”* (P15).

*“The pharmacist in my city is very competent. When I have a new prescription, he explains to me how to take it well. He has become a friend because I have been going to his pharmacy for so many years. It’s valuable.”* (P12).

- A negative perception of clinical research

Some patients had negative representations of clinical research (e.g., linked to the business generated by pharmaceutical companies (P04)), which affected their decision to participate in all kinds of research projects.

“*There is a lot of money in [research] (…). So I say to myself it's money, money and money*.” (P08).

#### How do patients who refused to participate in the PANDIA-IRIS study explain the way they manage their treatments at home?

- A rigorous medication management as described by patients at interview initiation…

All patients mentioned a well-established and ritualized medication management routine. Medication was often associated with daily activities (e.g., after getting up, mealtime, coffee time, and bedtime). Medications were stored in a dedicated place, and preparation of the weekly pillbox became routine. Some patients had implemented personal tools or techniques to avoid forgetting or taking their medication twice (e.g., notes, alarms, reminders from their partner, putting the medication in the jacket when going out, putting the medication box in a different place after taking them). Retired or non-working patients reported fewer constraints to taking their medications compared to the time when they were employed full-time.

“*What at first seemed so huge to me… It has calmed down, it has become easier. But it takes time anyway. I've been doing it [the weekly pillbox] for years and it suits me a lot. (…) I had my list, it was very complicated really, and little by little you get used to it, then you do it almost with your eyes closed” (P05).*

Self-confidence, known role, perceived needs, and importance of each drug have been reported as the main sources of motivation to adhere to treatment. Some patients reported a journey toward resilience and acceptance of long-term treatment. Some patients referred to their responsibility to take treatment, associated with a desire to live, and the fear of deterioration of the disease.

“*My life depends on [the medication]. My kidneys are still functioning, but I don’t know how long for (…). Preventing them [the kidneys] from declining motivates me to take my medication. Well, you can also live with dialysis but… if I can avoid it, I will do everything possible. (…) I want to be alive and I will take my medication in order to keep myself fit.”* (P06).

The interviewees were actively engaged in the management of their disease and described themselves as self-determined and empowered. Most of them read the medication leaflets, looked for information on the internet, or asked their physician, and sometimes their pharmacist, about side effects or medication interactions.

*“I'm a pro now. (…) Before I had to be more careful. I've always been able to manage it. (…) I'm autonomous, I manage my medication, I manage my illnesses, I think I know my body pretty well, I'm not a doctor but I know myself pretty well. And that's a lot.”* (P13).

Support from relatives also played an important role in this process. If a family caregiver was in charge of the patient's medication, he/she was systematically contacted to obtain his or her opinion on participation in a study. Among the interviewed patients, female spouses were often in charge of managing their husbands’ medication (e.g., filling the weekly pillbox or refilling medication at the pharmacy).

*“So I don't manage them [the medications], my wife does the work (…)* Patient's wife*: “And he wouldn't be able to prepare the weekly pillbox by himself.”* (P04).

- However, when asking open-ended questions throughout the interview, the patients had detailed difficulties in managing their treatment.

Interestingly, while the interview proceeded with open-ended questions about possible forgetfulness or misuse of medication, some patients expressed denial of their illness and banalization of nonadherence.

Patients reported a few instances of unintentional forgetting, delayed intakes, or voluntary omissions, which occasionally occurred because of a disrupted routine (e.g., appointments, social encounters). Episodes of confusion were described in the cases of medication or regimen changes. Some patients also explain that the high cost of treatment was a risk factor for nonadherence.

*“If I'm invited to a friend's house or if I go to a restaurant, it's true that I don't always take my insulin syringe (…). So I skip the [insulin] shot. (…) I can see that the blood sugar rises a bit more than usual, but well.” *(P02).

The transition from forgetting once in a while to not taking the treatment at all happens sometimes unintentionally, for example, in relation to a personal burden, as explained by the patient below:

*“It annoys me if one day I forget because I am so busy helping my husband (…) I remind him but nobody reminds me (…) My husband is also ill and under heavy treatment and he can't prepare his weekly pillbox alone, and I have to do everything. In addition to mine, it is a tsunami, it's a lot (…). The mental load of women has nothing to do with the mental load of men.”* (P05, caregiver burden).

When moving forward with the interview, patients were invited to talk about their medication and disease management throughout their therapeutic journey. The patients explained their concerns about the constant increase in the number of prescribed medications. Some participants talked about a “*cocktail*” of medications that was not easy to accept (P14, P15).

*“Over the years I finally resigned myself, I accepted it. At first, I was a bit upset every time my physician added a medication… I found it difficult to accept. However, I got used to the cocktail. (…) Well, I accepted it of course, but I thought it was a lot, adding one extra pill every eight or ten months. Well… I trusted him [the physician].” (P15).*

Hence, a few patients described their personal beliefs, ambivalence about their treatments, and fear of adverse effects.

*“We have more and more pills… We don’t know the role of each of them. This is frustrating that we don’t know if a pill interacts with another.”* (P04).

*“Diabetes has always been a problem for me. (…) At the beginning, I didn't take care of [the diabetes]. I didn't manage it properly. (…) I was taking my medication but there were a lot of things I couldn't stand (…) So it's true that I didn't really pay attention to that [the diabetes], and (…) my [blood sugar] levels were high.”* (P13).

*“I am against medication, of course I don't like to take medication for anything. (…) Of course I'd like not to take them (…). It's like a laboratory [when preparing the weekly pillbox].”* (P05).

*“I’ve been through this before two years ago. Yeah, it was boring, it was almost a constraint (…). I was pretty rebellious about the whole stinging thing and everything because, I think I’ve had enough”* (P09).

Patients described their emotions during their therapeutic journey as stress (P05, P09), fear (P05, P13), concern about disease degradation (P02, P03, P04, P06, P13), feeling overwhelmed about the disease burden and treatment complexity (P03, P04, P05, P09, P10, P12, P13), frustration in case of treatment ineffectiveness (P02, P11), and shame for not being able to manage the disease efficiently (P13).

#### What is the relationship between patients who refused to participate and their HCPs?

The relationships between the patient and HCPs were either complete trust or a difficult relationship. Some interviewees were engaged in regular shared-decision processes with their physicians (e.g., they asked for regimen adjustments according to efficacy and side effects).

*“I sometimes suggest my physician to change my medicines. For instance, I can't stand the medicines “Co-Lisinopril [lisinopril* + *hydrochlorothiazide]” in summer because I tend to sweat more and it causes low blood pressure! In winter, I could take the “Co-Lisinopril” and the “simple Lisinopril” in summer. I suggested this to my physician who accepted the proposal.”* (P02).

On the other hand, physicians’ lack of time and empathy were perceived as barriers to sharing information about medication management. Two patients explained that a breach of trust with the physician led to nonadherence, expressed either as a discontinuation of their treatment for several months or voluntary defective treatment implementation. Another barrier is physician turnover, which frequently occurs in university hospitals. This prevents patients from sharing their medication management if the relationship is renewed.

*“I had a problem with endocrinologists. (…) When we begin the consultation, they tell us: “show me your booklet!” I hate this booklet. I have it with me but I say I don't have it. I'm so afraid, because when we have high blood sugar concentrations, they say “you didn't pay attention” whereas I did. (…)”* (P13).

#### How do patients who refused to participate perceive the role of their HCPs about medication management support?

Patient confidence in physicians, pharmacists, and nurses is unequal. Physicians occupy a privileged place in the minds of patients to discuss medication management. For many patients, this was reinforced by accessibility of their medical records. According to one patient, nurses were too overloaded to talk about medication management, while another patient explained being less confident about talking to a nurse compared to a pharmacist or a physician. A patient’s wife (P04) believed that home care nurses were paramedical staff and could not be trusted.

“*Some people may have more difficulty to confide in a nurse compared to a physician because of their social status.*” (P06).

The patient’s perception of the pharmacist’s role was dichotomous. On the one hand, patients explained that the skills and availability of their pharmacists were appreciated and reassuring. The usefulness of pharmacy services, such as the preparation of weekly pillboxes, was praised (e.g., “*Making a weekly pillbox for them [patients who need it] is already a precious help*”. (P05)).

On the other hand, the pharmacist’s role seemed to be often perceived as technical (e.g., the role of the pharmacist is only to “*control*” the treatment. (P06)). Pharmacy is still perceived as a point of sale rather than a place of pharmaceutical care. The lack of confidentiality and the stand-up position at the pharmacy made the exchange less comfortable than in the doctor’s office (P14). The concept of continuity of care and interprofessionality was absent in most patients’ speech, and this influenced patients’ decisions to participate in the pharmacist-led intervention study.

*“I expect the pharmacist to advise me and to inform me about the compatibilities of the medicines. It will always be difficult for a pharmacist to replace the doctor, as he/she [the pharmacist] does not have access to the medical file, he/she does not know the case.”* (P02).

*“I never thought about pharmacists [to discuss about medication taking]. (…) My doctor tells me what to take.”*(P07).

*“The pharmacist can only repeat the doctor’s recommendations.”* (P08).

*“[My pharmacist] does not understand anything, we cannot discuss. I come to take my medicines to the pharmacy and that’s all, goodbye. This is totally different from my previous usual pharmacy, where we could discuss, laugh, it was nice. But now it is not the case anymore*.” (P08).

## Discussion

### Main results

This study addressed a common yet under-investigated issue—patients’ refusal to participate in an interprofessional medication adherence intervention study. Taken together, our PART-PANDIA results showed that more women than men refused to participate, and patients who refused had a longer diabetes experience than those who accepted. However, patients in both groups were clinically at risk, as their renal function continued to decrease over time at a faster rate than the usual diabetic population. More patients who accepted the PANDIA-IRIS study signed the GCF compared to patients who refused, showing that the included patients were more willing, available, and interested in research than non-participants. The participants also stressed the importance of tailoring the adherence program to fit their busy medical schedules and needs. The included patients found the intervention useful to improve their medication and disease management and sharpen their knowledge. They felt reassured by close monitoring and emerging interprofessional collaboration, especially through the adherence report shared between HCPs. By contrast, patients who refused did not see the point of being part of such an intervention, which they perceived to be a burden. Even though they described rigorous medication management, they also described adherence difficulties.

### Benefits of the intervention

By participating, the included patients discovered the usefulness of the intervention and became active partners. They described that improving their treatment management was a complex process, based on knowledge, learning attempts, individual efforts, and a safe environment to explore their beliefs about medication [29–31]. The common emotional burden of polymedicated patients is a theme shared by participants and non-participants, which should be addressed by interprofessional interventions.

For ethical reasons, few studies have compared medication adherence in adherence interventions among non-participants and participants. Therefore, it is difficult to understand if the level of medication adherence and acceptance of participation are correlated. Based on self-reports, non-participants reported significantly higher diabetes self-management compared to participants in an educational diabetes intervention [15]. Another study reported no difference in adherence between participants and non-participants to an adherence intervention for cardiovascular risk management, but only 36% were considered highly adherent in both groups [32]. A RCT  including patients with type 1 diabetes aimed at defining optimal treatment to prevent nephropathy and retinopathy complications. Patients who reported a high study burden had a lower adherence during the first year and a lower study retention compared to patients who reported lower barriers to participation [13].

### Patients at risk of non-participation

More women refused to participate than men. Even though the women described themselves during recruitment as autonomous in managing their treatment, they could trivialize their need for support, which may impact their own medication adherence (e.g., women reporting caregiving involvement and burden [13, 33, 34]). It is unclear whether gender is a risk factor for nonadherence as many other covariates are involved (e.g., perceived treatment side effects, age, and comorbidities) [35–39].

Patients who benefit from strong support from family caregivers are at risk of non-participation if an informal caregiver is absent when the study is presented to the patients. Such caregivers are essential to ensure optimal medication adherence [40] and special attention should be paid to this dyad during study presentations. Importantly, a supportive medication adherence program must be presented early enough during the care itinerary so that patients can get the full benefit of the intervention to reinforce their autonomy before having to rely on a family caregiver.

Patient recruitment in diabetes education programs is often low, whereas such programs are strongly recommended to achieve clinical outcomes and increase quality of life [41, 42]. The patients’ decision to participate in an intervention depends on multiple complex factors [17, 43], including logistical reasons or lack of personal interest [13, 17, 44]. Patients who refuse may not consider or understand the positive impact of the intervention on their medication management, medication literacy, and quality of life. Literature shows that this aspect has been encountered and cited as the “don’t know” factor and was a key barrier to participation in an educational program for patients with type 2 diabetes [14].

### The intervention should be offered sooner in patients’ therapeutic journey

The patients who refused to participate had a longer history of diabetes. These patients had a long treatment experience and may have acquired habits and self-confidence about their disease and treatment management over the years, which they relied on and felt reluctant to discuss. In our study, non-participants tended to be older than participants. In the study by Thoolen et al., patients treated intensively were more likely to participate in the first year after diabetes diagnosis than 2–3 years after [15]. Offering interventions for patients newly diagnosed with diabetes could increase the inclusion rate. In a meta-analysis including 771 adherence studies, the effects of the interventions on medication adherence outcomes seem to be smaller in older patients compared to younger [45]. Adherence interventions must be presented to patients at the right time according to a predefined shared plan to allow patients to actively participate in the decision with HCPs.  Indeed, medication adherence is a dynamic process which can drastically evolves over time, especially after major clinical or personal events (e.g., hospitalization, moving, giving birth, death of a close relative). Outside of a trial context, polypharmacy patients should first experience the IMAP for some months to obtain a reliable evaluation of their medication adherence before deciding whether they need to pursue an intervention or not. This would allow to make patients aware of the existence of such adherence programs so that they could participate upon their own perceived needs during their care itinerary. Based on the recruitment experience, we noticed that patients who refused could have been less at ease in discussing their treatment management difficulties and may have needed time to decide to participate. This should be taken into consideration when introducing the intervention step by step. To increase access to effective medication adherence interventions, patient participation should be planned in advance based on a shared-decision model.

### New models of care and interprofessionnality

The PART-PANDIA study demonstrated that a trusted relationship between the patient and HCPs plays an important role in patients’ decision to participate in an intervention, reinforcing what has already been described in the literature [14, 16, 46]. Many patients refuse to participate in medication adherence interventions that are not yet nested into routine care; such interventions may be perceived as extra-curricular, optional, and sometimes confronting. In our study, non-participants did not understand the role of the pharmacist in supporting medication adherence, showing a lack of information on the many interventions that the pharmacist can provide to support medication management. In addition, they prioritized their physician as the main contact person and the leader of their care, although the responsibility was shared interprofessionally between pharmacists and nurses. Patients may hierarchize HCPs through personal and social representations. The shared decision-making process with pharmacists is not well established; however, it is increasingly acknowledged between physicians and patients [47]. Therefore, patients should be better informed of the plurality of the roles and responsibilities of each HCP in monitoring medication management. This transition of care toward interprofessional collaborations, including the patient as a partner, may help improve medication adherence.

### Potential room for improvement of the adherence intervention

Patients’ reasons for refusal were often similar to the barriers cited by patients who left the study prematurely (e.g., too many appointments and EM being bulky). To overcome this problem, efforts must be undertaken to adapt interventions to patients’ needs (e.g., implementing tele-consultation to prevent disabled patients from coming regularly to the intervention site, adapting the tool to measure adherence). In addition, clinical research should be made more accessible and attractive, especially for vulnerable patients or those with low health literacy (e.g., those with low retention in care).

### Strengths and limitations

This study had several strengths, especially the robustness of its mixed quanti-quali methodologies. All sociodemographic and clinical variables were extracted from patients’ medical records -the most reliable information source- with few missing data. According to an adjusted multivariate analysis, we could identify that participants and non-participants differed in sex and time from diabetes diagnosis. The calculation of the eGFR decline is based on a rigorous methodology, which is clearly explained. This calculation is scarce in the current literature whereas it allows to characterize renal decline in DKD patients.

This study had some limitations. First, eligible patients recruited in the PANDIA-IRIS study who rejected GCF were not included in the quantitative analysis. We cannot exclude the possibility that patients who refused GCF differed from those who signed it. Nevertheless, two-thirds of the eligible patients were included in the PART-PANDIA study, providing a substantial database for analyzing demographic and clinical variables in both groups. Second, the patient’s education level was not available in the patient’s record, whereas literature shows that there could be a differential education level among participants compared with non-participants [15]. Third, as interviews with non-participants were hold several months after the recruitment date, a recall bias about their reasons for refusal cannot be excluded. However, this bias was limited as at the beginning of the interview, the investigator reminded the patients about the context of the recruitment, showed them the EM, and explained them again the study procedures and design. Forth, patients who refused to participate in the qualitative interviews would have brought up other themes, even though the recruitment was sufficiently heterogeneous to limit selection bias.

## Conclusions

To our knowledge, this is the first study to compare factors and reasons for participation and non-participation in a medication adherence intervention in patients with DKD using a mixed methods approach. Patient recruitment in educational diabetes programs is a difficult task, and patients who refuse to participate may not be aware of the benefits they could gain from their medication management, medication literacy, and quality of life. Understanding systematic factors and reasons for non-participation helps tailor intervention designs to the needs of such polypharmacy patients to facilitate their engagement in medication adherence interventions. Information on specific support in medication management that pharmacists can provide within an interprofessional healthcare team must be better promoted among chronically ill patients.

## Data Availability

The datasets generated and analysed during the current study are not publicly available due to ethical reasons but are available from the corresponding author on reasonable request.

## Abbreviations

BMI: Body Mass Index
CHUV: *Centre Hospitalier Universitaire Vaudois*, Lausanne University Hospital
CKD-EPI: Chronic Kidney Disease Epidemiology Collaboration formula
DKD: Diabetic Kidney Disease
eGFR: Estimated Glomerular Filtration Rate
EM: Electronic Monitor
ESRD: End stage Renal Disease
GCF: General Consent Form
HbA1c: Glycated Haemoglobin
HCP: Healthcare Provider
IQR: Interquartile Range
IMAP: Interprofessional Medication Adherence Program
KDIGO: Kidney Disease Improving Global Outcomes guidelines
LADA: Latent Autoimmune Diabetes in Adults
LCD: Liquid–crystal display
LDL: Low Density Lipoprotein
MDRD: Modification of Diet in Renal Disease
NSTEMI: Non-ST Elevation Myocardial Infarction
PANDIA-IRIS: *Patients Diabétiques et Insuffisants Rénaux: un programme interdisciplinaire de soutien de l’adhésion thérapeutique*
PART-PANDIA: PARTicipation to PANDIA-IRIS
RCT: Randomized Controlled Trial
RRT: Renal Replacement Therapy
SD: Standard Deviation
SRQR: Standards for Reporting Qualitative Research guidelines
STEMI: ST Elevation Myocardial Infarction
STROBE: STrengthening the Reporting of OBservational studies in Epidemiology guidelines
TIAs: Transient Ischemic Attacks
USA: United States of America
